# Long-term effect of using hard contact lenses on corneal endothelial cell density and morphology in ophthalmologically healthy individuals in Japan

**DOI:** 10.1038/s41598-023-34756-x

**Published:** 2023-05-11

**Authors:** Takashi Ono, Toshihiro Sakisaka, Keita Takada, Shota Tokuda, Yosai Mori, Ryohei Nejima, Takuya Iwasaki, Takashi Miyai, Kazunori Miyata

**Affiliations:** 1 Department of Ophthalmology, Miyata Eye Hospital, 6-3, Kuraharacho, Miyakonojo, Miyazaki, 885-0051 Japan; 2 Department of Ophthalmology, Graduate School of Medicine, The University of Tokyo, Tokyo, Japan

**Keywords:** Eye manifestations, Paediatric research, Medical research, Outcomes research

## Abstract

The adverse effects of hard contact lenses (HCL) on the corneal endothelium have been studied in the short term; however, long-term effects remain still unclear. In this study, we analyzed the effect of long-term HCL use on corneal endothelial cell density (ECD) and morphology in healthy Japanese individuals. This cross-sectional observational study included individuals using HCL for refractive errors examined at a single specialty eye hospital. Patient age, duration of HCL usage, ECD, coefficient of variation of the cell area (CV), and rate of appearance of hexagonal cells (6A) obtained via non-contact specular microscopy were assessed. We analyzed 8604 eyes (mean age: 35.6 ± 10.0 years, 837 males, 3465 females). The mean duration of HCL usage was 14.7 ± 9.1 (range, 1–50) years. Multivariate analysis revealed that ECD significantly correlated with age (*P* < 0.001) but not with duration of usage; however, CV and 6A significantly correlated with both factors (*P* < 0.001). Univariate analysis revealed that CV and 6A correlated with duration of usage (all, *P* < 0.001). According to our results, CV and 6A correlated with the duration of HCL usage in ophthalmologically healthy Japanese individuals. Therefore, it is important to monitor corneal endothelial morphology in long-term HCL wearers.

The clinical use of hard contact lenses (HCL) in the eye as a method of refractive correction is widespread. The development of new materials with high oxygen permeability in HCL has improved the usability of rigid gas-permeable (RGP) lenses. Soft contact lenses, which are more comfortable, are currently replacing HCL for myopic correction. Nevertheless, HCL continues to be clinically important for pediatric patients with myopia^1,2^ and patients with keratoconus^3,4^, aphakia^5^, and strong corneal astigmatism that cannot be corrected with glasses or soft contact lenses^6,7^. The adverse effects of HCL on the corneal endothelium have been studied for many years, and short- or medium-term observations have shown that the size and shape of the endothelium is affected^8^. However, very few studies have demonstrated the long-term effects on the cornea^3,4,9^.

The corneal endothelium is made up of hexagonal cells located inside the cornea that maintains transparency through barrier and pump functions^10^. Long-term use of contact lenses affects the condition of corneal endothelial cells and can impair normal endothelial function and visual acuity. Therefore, patients with impairment of the corneal endothelium should refrain from using contact lenses, because it can cause corneal edema and greatly reduce visual function. The parameters of normal corneal endothelium vary among population subsets worldwide^11–18^, and the clinical effect of HCL on the corneal endothelium may differ depending on the population. Although the morphology of the corneal endothelium is affected by the usage of HCL^19,20^, no study has investigated these factors in a Japanese population. Furthermore, studies evaluating the effect of HCL use among ophthalmologically healthy populations are limited in terms of number and duration of usage because patients without ophthalmic diseases are not frequently assessed for corneal endothelium morphology. Therefore, we aimed to investigate the effect of long-term HCL use on corneal endothelial cell density (ECD), polymegathism, and pleomorphism in healthy Japanese individuals.

## Results

A total of 8604 eyes of 4302 patients (male:female = 837:3465) were included in the study. The mean age of the patients was 35.6 ± 10.0 years, and mean duration of HCL usage was 14.7 ± 9.1 (range, 1–50) years. Demographic data of the patients are summarized in Table 1.

**Table 1:** Demographic background of users of hard contact lens.

	Total	Male	Female	*P*-value
N (eyes)	8604	1674	6930	–
Age (years)	35.6 ± 10.0	35.3 ± 9.7	35.7 ± 10.1	0.15
ECD (cells/mm^2^)	2907.6 ± 335.8	2909.9 ± 322.4	2907.1 ± 338.9	0.86
CV (%)	33.3 ± 7.2	33.4 ± 7.4	33.3 ± 7.2	0.68
6A (%)	57.0 ± 10.6	57.0 ± 10.7	57.0 ± 10.5	0.89
Cell area (μm^2^)	353.4 ± 41.7	353.6 ± 38.9	353.3 ± 42.4	0.81
Usage period of hard contact lens (years)	14.7 ± 9.1	13.2 ± 8.5	15.1 ± 9.2	< 0.001

Regarding the types of HCL used, exclusive usage of polymethyl methacrylate (PMMA) lenses was observed in 36 eyes of 18 patients, low-Dk (oxygen permeability) lenses (Dk < 60) in 714 eyes of 357 patients, and high-Dk lenses (Dk ≥ 60) in 820 eyes of 410 patients. The other patients (7034 eyes) used multiple types of HCL, and the period of usage was unknown, or information regarding lenses obtained during the interview was insufficient. The low-Dk group included 65 males and 292 females with the mean age of 35.7 ± 9.3 years, and high-Dk group comprised 103 males and 307 females with the mean age of 31.6 ± 8.2 years. Based on stratification of data by the decade of use, the endothelial parameters of patients who used HCL for 1–9, 10–19, 20–29, 30–39, and ≥ 40 years are summarized in Table 2.

**Table 2:** Parameters of the corneal endothelium in long-term users of hard contact lens.

Usage period of hard contact lens (years)	1–9	10–19	20–29	30–39	40–
N (eyes)	2504	3268	1962	782	88
Age (years)	27.5 ± 8.0	33.4 ± 6.2	42.6 ± 5.2	50.7 ± 4.8	60.0 ± 3.3
ECD (cells/mm^2^)	3009.8 ± 320.6	2930.6 ± 314.7	2815.9 ± 328.8	2739.6 ± 341.4	2684.8 ± 416.9
CV (%)	30.7 ± 6.1	33.3 ± 6.5	35.4 ± 7.8	37.3 ± 8.2	37.9 ± 8.3
6A (%)	60.1 ± 11.2	56.6 ± 10.2	54.9 ± 9.7	53.5 ± 9.3	52.6 ± 7.8
Cell area (μm^2^)	340.8 ± 37.1	350.5 ± 38.8	361.4 ± 42.2	371.1 ± 45.6	385.4 ± 55.4

**Figure 1:**
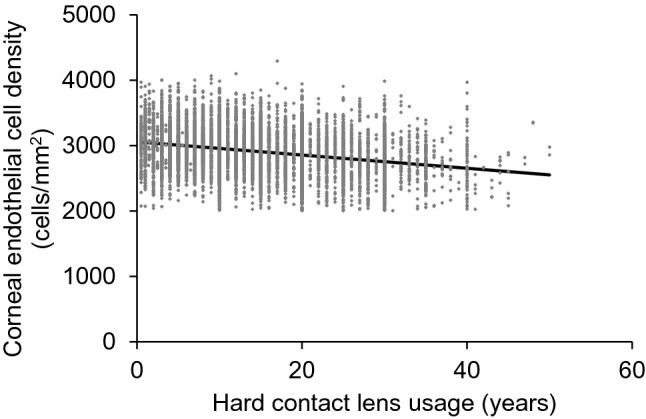
Effect of hard contact lens usage on corneal endothelial cell density. Corneal endothelial cell density is significantly related to the hard contact lens usage based on the linear regression model (Y = -10.12X + 3056.77, R^2^ = 0.075, *P* < 0.001).

**Figure 2:**
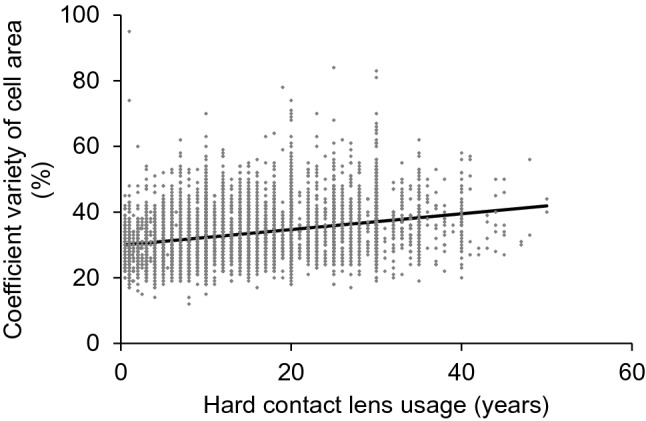
Effect of hard contact lens usage on the coefficient of variation of the cell area. The coefficient of variation of the cell area is significantly related to the hard contact lens usage based on the linear regression model (Y = 0.24X + 29.87, R^2^ = 0.094, *P* < 0.001).

**Figure 3:**
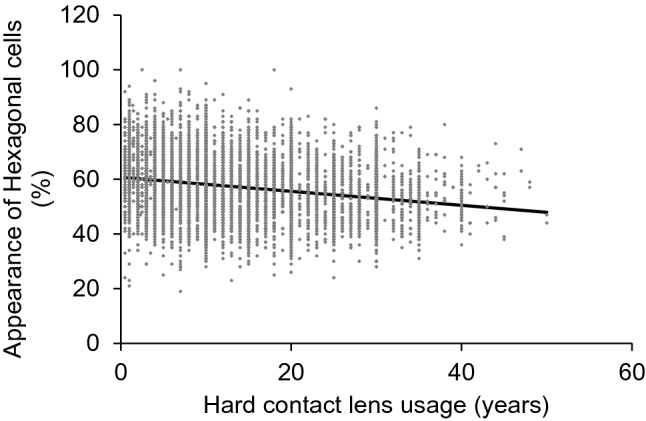
Effect of hard contact lens usage on the appearance of hexagonal cells. The appearance of hexagonal cells is significantly related to the hard contact lens usage based on the linear regression model (Y = -0.26X + 60.71, R^2^ = 0.050, *P* < 0.001).

**Figure 4:**
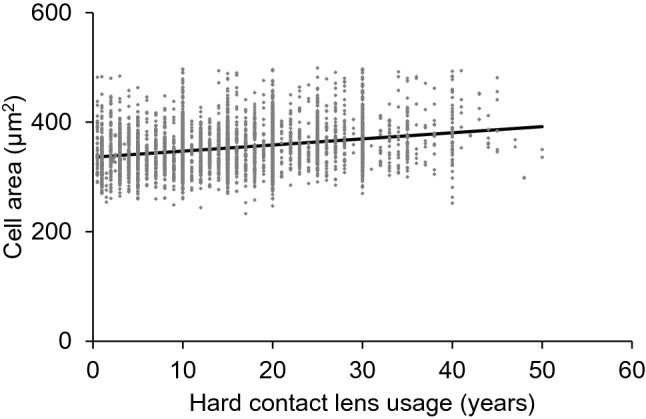
Effect of hard contact lens usage on the cell area of corneal endothelium The corneal endothelial cell area is significantly related to the hard contact lens usage based on the linear regression model (Y = 1.12X + 336.06, R^2^ = 0.068, *P* < 0.001).

Univariate analysis demonstrated that ECD was significantly decreased in association with the duration of HCL usage (Y =  –10.12X + 3056.77, R^2^ = 0.075, *P* < 0.001, Fig. 1). Statistical analysis also revealed that the coefficient of variation of the cell area (CV) (Y = 0.24X + 29.9, R^2^ = 0.094, *P* < 0.001, Fig. 2), rate of appearance of hexagonal cells (6A) (Y = –0.26X + 60.7, R^2^ = 0.050, *P* < 0.001, Fig. 3), and cell area (Y = 1.1X + 336, R^2^ = 0.068, *P* < 0.001, Fig. 4) correlated significantly with the duration of HCL usage. In the sub-group analysis using a linear regression model based on the oxygen permeability of lens, ECD, CV, 6A, and cell area were significantly associated with the duration of HCL usage in the low- and high-Dk groups (Table 3). ECD change per one-year of HCL usage in the low-Dk group was −5.42 (95% confidence interval, from −6.88 to −3.96) cells/mm^2^ and −5.05 (95% confidence interval, from −6.64 to −3.45) cells/mm^2^ in the high-Dk group.

**Table 3:** Relationship between usage period of hard contact lens and corneal parameters in low- and high-Dk groups.

	Low-Dk group				High-Dk group			
	Regression coefficient	Lower 95% CI	Upper 95% CI	*P*-value	Regression coefficient	Lower 95% CI	Upper 95% CI	*P*-value
ECD (cells/mm^2^)	−5.42	−6.88	−3.96	< 0.0001	−5.05	−6.64	−3.45	< 0.0001
CV (%)	0.21	0.15	0.27	< 0.0001	0.088	0.054	0.12	< 0.0001
6A (%)	−0.24	−0.32	−0.15	< 0.0001	−0.18	−0.23	−0.12	< 0.0001
Cell area (μm^2^)	0.47	0.11	0.83	< 0.0001	0.35	0.16	0.55	< 0.0001

*ECD* endothelial cell density; *CV* coefficient of variation of the cell area; *6A* rate of appearance of hexagonal cells.

Next, we performed multivariate analysis to consider the effect of age-related changes in ECD and found that ECD was significantly correlated with age (*P* < 0.001), but not with the duration of usage; however, CV and 6A correlated significantly with both factors (all *P* < 0.001, Table 4). Additionally, in our study population, male sex and older age were significantly related to ECD (both P < 0.001) and CV (*P* = 0.04 and < 0.001, respectively). Older age was also significantly related to 6A (*P* < 0.001) (Table 4).

**Table 4:** Results of the multivariate analysis of corneal endothelial parameters.

Objective variable	Explanatory variable	Partial regression coefficient	Standardized partial regression coefficient	*P*-value
ECD	Usage period of hard contact lens (years)	−1.07	−0.029	0.07
	Sex (Male)	−3.55	−0.0042	< 0.001
	Age (years)	−10.69	−0.32	< 0.001
	Constant values	3304.91	–	–
CV	Usage period of hard contact lens (years)	0.16	0.21	< 0.001
	Sex (Male)	0.40	0.022	0.04
	Age (years)	0.092	0.13	< 0.001
	Constant values	27.65	–	–
6A	Usage period of hard contact lens (years)	−0.18	−0.15	< 0.001
	Sex (Male)	−0.37	−0.014	0.21
	Age (years)	−0.097	−0.093	< 0.001
	Constant values	63.04	–	–

*ECD* endothelial cell density; *CV* coefficient of variation of the cell area; *6A* rate of appearance of hexagonal cells.

## Discussion

We examined the density and morphology of corneal endothelial cells in more than 8000 eyes in patients using HCL for an extended period of more than 14 years. Using multivariate analysis, we demonstrated that the duration of usage is not related to ECD but is related to CV and 6A. HCL usage leads to corneal hypoxia, which in turn leads to lactate accumulation, increased CO_2_ concentration, and changes in the pH of the cells^21,22^. These homeostatic changes in cellular environment result in morphological changes, polymorphism (decreased in 6A), and polymegathism (increased CV). Previous reports showed that polymegathism occurred after long-term usage of HCL^19,20^. Furthermore, mechanical stimulation of the corneal surface while using a contact lens reportedly leads to the release of inflammatory mediators in tear samples^23^. Moreover, ultraviolet (UV) radiation can affect the corneal endothelium^24^. Therefore, the protective effect of HCL against UV might be related to this phenomenon. However, our retrospective data did not include the types of HCL and did not consider whether they had UV protection. Detailed elucidation of the effect of HCL on the corneal endothelium is required, and future analysis and comparison should be performed for basic research.

In this study, changes were observed in the corneal endothelial cell morphology, whereas ECD did not change. It has been reported that ECD does not change with contact lens usage and the results of this study are consistent with those of previous reports^25,26^. Since the present study included individuals who had used HCL for extended periods, it was necessary to consider age-related changes in the endothelium. While univariate analysis showed that ECD had changed, multivariate analysis considering the effect of age demonstrated that the number of years of HCL usage had no direct effect on ECD. ECD gradually decreases with aging in both young and older adults^17^. Although corneal endothelial cell morphology also changes with age, even after accounting for these changes in the statistical analysis, long-term use of HCL is related to 6A and CV of the endothelium. Unlike the medium-term perspective, this study examined cases of long-term use with an average of more than 14 years, and also included cases in which HCL had been used for more than 40 years; therefore, these conclusions can be considered to be reliable.

HCL is commonly classified into PMMA and RGP lenses. PMMA lenses, which have low oxygen permeability, reportedly result in contact-lens-induced lactate accumulation, changes in pH, CO_2_ elevation, and polymegathism^21^. The RGP lens was developed to improve gas permeability (high Dk value), and it induced fewer morphological changes in the corneal endothelium, but morphological changes were still observed^27,28^. The effect of the lens type on the condition of the corneal endothelial cells may differ. In this study, we considered the Dk value of 60 as a threshold value. Our results showed that ECD decrease per one-year of HCL usage was -5.42 cells/mm^2^ and -5.05 cells/mm^2^ in the low- and high-Dk groups, respectively. This change, which includes age-related changes, was not high considering the normal age-related range for Japanese patients^17^. Changes in CV tended to be lower in the high-Dk group than in the low-Dk group (0.21% vs. 0.088%). Although HCL made of PMMA are not widely prescribed now, there are still patients who have been using the same familiar lenses for a long time. Therefore, the effect of these lenses on the cornea cannot be ignored. It is important to fully understand the changes in morphological characteristics as well as the ECD, and frequent follow-up is necessary, especially in cases where corneal endothelial cells are damaged by ocular trauma, surgical intervention, or other disorders.

Endothelial morphological abnormalities are thought to be caused by changes in the physiological environment, such as cellular hypoxia of the corneal endothelium and systemic diseases, such as diabetes^29,30^. This environmental stress induces changes in the CV and 6A, leading to a decrease in ECD. However, our multivariate analysis showed that contact lens use was not significantly associated with ECD, but with endothelial morphology. A previous study showed the same results as ours, and their multiple regression analysis showed that contact lens use was not significantly related to ECD, but to endothelial morphology (CV), but this report was based on a small number of patients, and hard or soft lenses and the type of contact lens was not classified^31^. One reason why ECD is not affected by the duration of HCL usage is the cellular ability of the corneal endothelium to recover from such environmental stresses. Corneal endothelial cells have the ability to recover when the stress is removed or reduced^32^; HCL users typically do not use lenses throughout the day and remove them at night. This recovery period explains why ECD is not affected, and morphological changes only occur with HCL use. Clinically, it is important to observe the corneal state even after discontinuation of HCL usage.

In our current study, multivariate analysis revealed that male sex was significantly related to the ECD and CV in eyes with HCL. We previously reported that CV in ophthalmologically healthy males was lower than that in females in a Japanese population^17^. Although endocrine disorders, such as acromegaly, are related to ECD^33^, the relation of the state of corneal endothelium and sex difference is not fully analyzed and the issue is still controversial; therefore, a further comparative clinical study was required.

One limitation of this study is that the type of lens used by each patient was not available since many patients who had worn HCL for decades did not know the exact type of lens they used or how long they had been using them. However, interviews for the duration of usage were reviewed retrospectively and it was possible to perform a sub-analysis. Secondly, it was difficult to accurately calculate the total time of HCL use in this study, because it was not possible to determine the duration of HCL usage per day. Furthermore, this study was conducted at a single hospital, and we did not evaluate the degree of myopia and did not include a control group. To overcome this limitation, future studies should prospectively evaluate longitudinal data on the corneal endothelium in multiple settings.

In conclusion, long-term use of HCL causes morphological changes in the corneal endothelium, although it does not affect ECD. Clinical ophthalmologists should regularly monitor the morphological changes of the corneal endothelium as well as various complications of HCL.

## Methods

This retrospective cross-sectional study was approved by the Institutional Review Board of Miyata Eye Hospital (Miyazaki, Japan) (Identifier: CS-353-052). The study adhered to the tenets of the Declaration of Helsinki. Informed consent was obtained from all the participants through the opt-out method.

We included individuals who had used HCL (PMMA or RGP lens) for more than a year for refractive correction and whose corneal endothelium was evaluated at Miyata Eye Hospital from 1996 to 2015. We only included individuals whose bilateral eyes were accurately evaluated. We excluded patients who had used any soft contact lens and those with a history of ophthalmologic diseases, such as keratoconus or corneal transplantation, other than refractive errors. Medical charts were reviewed for age, ECD, 6A, and CV. CV and 6A were used to evaluate polymegathism and pleomorphism of the corneal endothelium. Information was also obtained on the type of contact lens based on products, such as PMMA and the Dk value (low Dk value < 60; high Dk value ≥ 60) from the patients’ interview.

Data about the corneal endothelium was obtained using non-contact specular microscopy (FA-3509, SP-8000, CA-2308, Konan, Nishinomiya, Japan), as reported previously^17^. Images of the corneal endothelial cells at the center of the cornea were captured, and all the endothelial cells within an area of 0.24 × 0.4 mm^2^ in the captured image were automatically traced, and ECD (cell/ mm^2^) was determined as described previously^17^. Simultaneously, 6A, CV, and cell area were also evaluated from the image using a program set in non-contact specular microscopy.

For statistical analysis, a linear regression model was applied to determine the relationship between the duration of HCL usage and ECD, 6A, CV, and cell area. The unpaired t-test was used for comparison between male and female participants. Multivariate linear regression analyses were used to determine the correlations between corneal endothelial parameters (ECD, CV, and 6A) and the duration of HCL usage, sex, and age. Cell area was not evaluated because it clinically reflects reciprocal numbers of ECD. Statistical analysis was performed using GraphPad Prism (version 9.0; GraphPad Software, La Jolla, CA, USA). All data are expressed as mean ± standard deviation unless otherwise mentioned. Statistical significance was defined as a two-tailed *P*-value of < 0.05.

## Data Availability

The data supporting the findings of this study are available upon request from the corresponding author (T.O.). The data are not publicly available because they contain information that can compromise the privacy of the research participants.
